# PATTERNS OF WEALTH TRAJECTORY IN LATER LIFE: CRITICAL PERIOD, ACCUMULATION, AND SOCIAL MOBILITY MODELS

**DOI:** 10.1093/geroni/igz038.1403

**Published:** 2019-11-08

**Authors:** Yu-Chih Chen, Sojung Park, Nancy Morrow-Howell

**Affiliations:** 1 Washington University in St. Louis, St. Louis, Missouri, United States; 2 Brown School, Washington University in St. Louis, St. Louis, Missouri, United States; 3 Washington University, St. Louis, Missouri, United States

## Abstract

Wealth, an important financial cushion for older adults to buffer economic stress, requires a longer time to accumulate and develop in one’s course of life. However, little is known about the trajectories of wealth in later life, and how the life course socioeconomic status (SES) may contribute to the development of wealth at old-age. This study investigated longitudinal patterns of wealth trajectory and whether SES across the life course affects these trajectories using critical period, accumulation, and social mobility models. Using data from 16,189 adults aged 51 and older from the 2004-2014 Health and Retirement Study, a growth mixture model was used to explore distinct wealth trajectories. Impacts of life course models were studied using multinomial logistic regression. Results showed that four heterogeneous latent classes of wealth were identified: Stable high (reference group), Low and increasing, Stable low, and High but decline. Disadvantaged adulthood SES, accumulated exposure to socioeconomic risks, and downward or persistent socioeconomic disadvantage over the life course were associated with Stable low, Low and increasing, and High but decline, supporting all three life course mechanisms on wealth development in later life. Evidence suggests that wealth development is heterogeneous across individuals, and a strong gradient effect of life-course SES on wealth trajectories are clearly observed. Programs and policies should address the effects of life course on wealth development to strengthen the economic well-being in later life.

